# The Mediating Role of Spiritual Intelligence in the Relationships Among Well‐Being, Professional Self‐Concept and Empathy Among Nurses

**DOI:** 10.1002/nop2.70613

**Published:** 2026-05-21

**Authors:** Ahmad Rayan, Suhair Al‐Ghabeesh, Manal Hassan Baqeas, Ibrahim Alenezi, Mirna Fawaz, Mohamad El Masri

**Affiliations:** ^1^ Faculty of Nursing Zarqa University Zarqa Jordan; ^2^ Faculty of Nursing Al‐Zaytoonah University of Jordan Amman Jordan; ^3^ College of Nursing Northern Border University Arar Saudi Arabia; ^4^ Faculty of Health Sciences, Nursing Department Beirut Arab University Beirut Lebanon

**Keywords:** empathy, professional self‐concept, spiritual intelligence, well‐being

## Abstract

**Aim:**

This study examined the potential mediating role of spiritual intelligence in the relationships among well‐being, professional self‐concept and empathy among nurses.

**Design:**

A descriptive cross‐sectional study design was adopted.

**Methods:**

The study included 143 nurses from one university hospital in Lebanon. Structural equation modelling (SEM) was used to test the hypothesized mediation model involving well‐being, professional self‐concept, empathy and spiritual intelligence.

**Results:**

Spiritual intelligence showed moderate positive correlations with well‐being (*r* = 0.53, *p* < 0.01), professional self‐concept (*r* = 0.54, *p* < 0.01) and empathy (*r* = 0.51, *p* < 0.01). Well‐being also correlated positively with professional self‐concept (*r* = 0.42, *p* < 0.01) and empathy (*r* = 0.52, *p* < 0.01). Professional self‐concept showed a positive correlation with empathy (*r* = 0.43, *p* < 0.01). Path analysis suggested that spiritual intelligence may play a mediating role in these associations (*p* < 0.01).

**Conclusion:**

Spiritual intelligence shows significant associations with pathways connecting professional self‐concept and well‐being to nurses' empathy. These findings support the development of targeted interventions and training programmes to strengthen spiritual intelligence in healthcare professionals, potentially fostering positive work environments and improved patient outcomes.

**Patient or Public Contribution:**

No Patient or Public Contribution.

## 1 Introduction

Spiritual intelligence is the capacity to access higher meanings, values and transcendent purposes, thereby maintaining inner and outer peace regardless of circumstances. Spiritual intelligence has been identified as an important coping resource during health crises, aiding recovery and care provision (Alrashidi et al. 2022). For example, during avian influenza and Ebola outbreaks, it promoted positive emotions, cooperation and effective patient management (Marshall and Smith 2015).

Individuals often turn to spiritual resources during times of conflict to cope with grief and loss. However, if an individual's sense of self is compromised, psychological instability may result, leading to feelings of isolation, despair and a lack of purpose (Abdolkarimi et al. 2022). Nurses with higher levels of spiritual intelligence may demonstrate enhanced competencies, self‐efficacy, communication skills and patient‐centeredness. Thus, spiritual intelligence is essential for nurses' career and personal development (Arad et al. 2022).

Well‐being refers to an individual's capacity to manage physical, psychological and social challenges using available personal and environmental resources (Kumar 2020). Well‐being is conceptualized from a holistic perspective encompassing energy, anxiety, positive mood and negative affect (pessimism). However, during epidemics and healthcare crises, the well‐being of frontline workers is often overlooked. Occupational stress may compromise healthcare professionals' well‐being and hinder professional goal attainment (Ta'an et al. 2020). Nurses frequently experience workplace stress, which may hinder goal attainment and professional development. Past studies have shown a positive relationship between an individual's well‐being and their spiritual intelligence (de Diego‐Cordero, et al. 2022).

Professional self‐concept refers to nurses' perceptions of their abilities and identity within the nursing role, as measured by the Nurse Self‐Concept Questionnaire (NSCI) across four domains: care, knowledge, staff relations and leadership. A weak professional self‐concept—shaped by education, professional image, ethics and sociocultural factors (Al Manaseer et al. 2025; Zhou et al. 2022)—increases risks of burnout, bullying, reduced job satisfaction and compromised patient safety (Farčić et al. 2020). Conversely, a strong professional self‐concept enhances clinical decision‐making.

Empathy is a multifaceted cornerstone of therapeutic nurse–patient relationships and clinical competence, comprising cognitive (perspective‐taking) and emotional (feeling‐sharing) components. Emotional empathy refers to the capacity to experience others' feelings while cognitive empathy denotes an individual's capability to understand and reconvey others' emotions (Matsuhisa et al. 2021). Empathy in healthcare is associated with several positive outcomes, including higher patient satisfaction, improved diagnostic accuracy, reduced patient distress, enhanced service quality and better treatment outcomes (Moreno‐Poyato et al. 2021).

Prior studies identified associations between spiritual intelligence (SI) and nurses' well‐being (Alrashidi et al. 2022), empathy (Aliabadi et al. 2021) and professional self‐concept (Hojat and Badiyepeymaiejahromi 2021; Farag et al. 2025). Despite heavy workloads, nurses must cultivate spiritual intelligence, well‐being, professional self‐concept and empathy to support ethical patient care. However, no research has tested an integrated model where SI mediates relationships among well‐being, professional self‐concept and empathy simultaneously in nurses. This study addresses this gap by testing an SEM‐based model among Lebanese hospital nurses, informing staff selection and spiritual intelligence training programmes.

### 1.1 Conceptual Framework

Spiritual intelligence (SI), based on King's (2008) model, encompasses critical existential thinking, personal meaning production, transcendental awareness and conscious state expansion. These capacities enable individuals to construct meaning, regulate internal experiences and align actions with higher values, making SI a key psychological resource in healthcare contexts (King and DeCicco 2009). In this study, SI is conceptualized as a mediating mechanism linking intrapersonal factors (well‐being and professional self‐concept) to interpersonal outcomes (empathy). First, well‐being provides the emotional and cognitive foundation for effective functioning; however, its influence on interpersonal care is not automatic. SI enhances well‐being by promoting meaning‐making and adaptive coping, which buffer stress and foster psychological balance (Sahebalzamani et al. 2013). In turn, SI enables individuals to transform this internal stability into purposeful, value‐driven engagement with patients. Second, professional self‐concept reflects nurses' perceptions of their competence, identity and role within clinical practice. SI strengthens self‐concept by fostering self‐awareness, existential purpose and alignment with professional values, thereby enhancing confidence and reducing burnout (Hojat and Badiyepeymaiejahromi 2021). Through this process, SI translates internal professional identity into meaningful caregiving behaviours. Finally, empathy is conceptualized as the relational outcome of these processes. Beyond emotional resonance, empathy in nursing involves cognitive understanding and compassionate action. SI contributes to empathy by facilitating transcendental awareness and connectedness, enabling nurses to respond to patients in a holistic and value‐based manner (King et al. 2012). Taken together, this framework proposes that SI functions as a central integrative pathway through which well‐being and professional self‐concept are transformed into empathic practice. This conceptualization justifies the hypothesized mediation model tested in the present study.

### 1.2 Aim

The aim of this study was to determine the mediating effect of spiritual intelligence in the relationships among well‐being, professional self‐concept and empathy among nurses.

## 2 Methods

### 2.1 Design

This study employed a cross‐sectional design to examine the mediating effect of spiritual intelligence in the relationships among well‐being, professional self‐concept and empathy among nurses from multiple clinical units.

### 2.2 Setting

This study was conducted across multiple clinical units (ICU/CCU, medical/surgical floors, cardiac care, ER and oncology) at a 158‐bed university hospital in Lebanon. The facility provides comprehensive health services with advanced capabilities, featuring private patient rooms equipped with modern monitoring technology.

### 2.3 Subjects

The study recruited full‐time nurses working across multiple clinical units (ICU/CCU, medical/surgical floors, cardiac care, ER, oncology) with at least 6 months' experience.

## 3 Instrumentation

### 3.1 Spiritual Intelligence Questionnaire

King's Spiritual Intelligence Questionnaire (2008) includes 24 items across four subscales: critical existential thinking, personal meaning production, transcendental awareness and conscious state expansion. Each item is rated on a 5‐point Likert scale (0 = Not at all true to 4 = Completely true), with all items summed except item 6, which is reverse scored. Total scores range from 0 to 96, where higher scores indicate greater spiritual intelligence. The questionnaire demonstrated strong reliability (Cronbach's alpha = 0.88) in previous studies (Hosseinjari and Zakeri 2010; Khodabakhshi Koolaee et al. 2019; King and DeCicco 2009).

### 3.2 Well‐Being Questionnaire

The Well‐Being Questionnaire (WBQ; Bradley 1994), specifically the 22‐item version (W‐BQ22), assessed general well‐being across four subscales: Depression (6 items), Anxiety (6 items), Positive Well‐Being (6 items) and Energy (4 items). Items used a 4‐point Likert scale (0 = Not at all to 3 = All the time); subscale scores were reversed where needed (e.g., Depression/Anxiety items) and summed for total WBQ score. Higher total scores indicate better well‐being. In this study, it demonstrated excellent reliability (*α* = 0.87), consistent with prior reports (*α* = 0.82–0.90; e.g., Alvani et al. 2015; Pouwer et al. 2000; Riazi et al. 2006; Savli and Sevinc 2005) across diverse populations, including Middle Eastern samples.

### 3.3 The Jefferson Scale of Empathy (JSE)

The Jefferson Scale of Empathy–Health Professionals version (JSE‐HP) was used to measure healthcare‐related empathy (Hojat et al. 2001). The instrument consists of 20 items rated on a 7‐point Likert scale (1 = strongly disagree to 7 = strongly agree), with total scores ranging from 20 to 140; higher scores indicate greater empathy. The scale comprises three subscales: perspective taking (10 items), compassionate care (8 items) and standing in the patient's shoes (2 items). The JSE has demonstrated strong psychometric properties across diverse cultural contexts, with reported internal consistency coefficients ranging from *α* = 0.87 to 0.94 (Williams and Beovich 2020). In Lebanon, the JSE has been applied among nurses and healthcare professionals, showing good reliability (Cronbach's *α* > 0.75) and supporting the original three‐factor structure (El Jakal et al. 2025). In this study, it demonstrated excellent reliability (*α* = 0.89).

### 3.4 Nurse Self‐Concept Questionnaire (NSCI)

The NSCI (Zencir et al. 2019) assesses professional self‐concept across its four domains—care, knowledge, staff relations and leadership—using 14 positively worded items rated on an 8‐point Likert scale (1 = definitely false to 8 = definitely true). Scores are averaged per domain for comparability. Validity and reliability were confirmed in Zencir et al.'s (2019) Turkish study, with Cronbach's alpha ranging from 0.83 to 0.91. The Persian version also showed strong reliability, with Spearman‐Brown and Cronbach's alpha coefficients of 0.84 and 0.97, respectively, and demonstrated good construct validity through significant inter‐subscale correlations (Badiyepeyma et al. 2013). In this study, it demonstrated excellent reliability (*α* = 0.91).

## 4 Data Collection

Ethical approval for this study was obtained from the Institutional Review Board (IRB) at Beirut Arab University and the participating university hospital. The informed consent of the nurses who were eligible to participate in the study was obtained, and the purpose of the study was explained to them before their participation. The participation was voluntary and anonymous; the questionnaires were given to the participants via the nursing office in a sealed envelope. The survey questionnaires were filled out and returned to the researchers in closed envelopes. Participants were given 10 days to complete and return the questionnaires.

### 4.1 Ethical Considerations

Ethical considerations were carefully addressed throughout the study. Approval to conduct the research was obtained from the responsible authorities. Informed consent was secured from all participating nurses prior to data collection. The anonymity of study subjects was strictly maintained, as no names or identifying information were recorded during data collection or reporting. Furthermore, all data obtained were kept confidential and securely stored to protect participant privacy.

### 4.2 Data Analysis

Data were analysed using the IBM SPSS version 27. The demographic information was presented using descriptive statistics. Correlation and multiple regression analyses were conducted to examine relationships among spiritual intelligence, well‐being, professional self‐concept and empathy. The significance level was set at 0.05 for each value. Structural Equation Modelling (SEM) was performed using AMOS version 27 with maximum likelihood estimation (ML). The mediation model tested direct paths (well‐being/professional self‐concept → empathy) and indirect paths mediated through spiritual intelligence. Standardized (*β*) and unstandardized (*B*) path coefficients, standard errors (SE), 95% CIs and *p*‐values were estimated and reported.

## 5 Results

### 5.1 Participant Characteristics

The sample comprised 143 nurses, with 108 (75.5%) female, 132 (92.3%) aged 21–29 years, 130 (90.9%) having 1–3 years experience, 127 (88.8%) muslim, 59 (41.3%) employed in ICU and 130 (90.9%) holding bachelor's degrees (Table 1).

**TABLE 1:** Sample characteristics.

Variable	Category	*N*	%
Gender	Male	35	24.5
	Female	108	75.5
Age	21–29 years	132	92.3
	30–39 years	8	5.6
	40–49 years	1	0.7
	50–59 years	1	0.7
	Older than 59 years	1	0.7
Years of Experience	1–3 years	130	90.9
	3–6 years	4	2.8
	6–10 years	7	4.9
	More than 10 years	2	1.4
Religion	Muslim	127	88.8
	Christian	9	6.3
	Druze	5	3.5
	Pagan	1	0.7
	Hindu	1	0.7
Work unit	Medical floor	20	14.0
	Surgical floor	15	10.5
	ER	7	4.9
	ICU	59	41.3
	Palliative care	5	3.5
	Geriatric	6	4.2
	Cardiac care unit	29	20.3
	Oncology floor	2	1.4
Educational level	BT	7	4.9
	LT	4	2.8
	BS	130	90.9
	MS	2	1.4

### 5.2 Scores of the Main Study Variables

Participants reported relatively high mean scores for spiritual intelligence (2.40 ± 0.63, 95% CI: 2.29–2.50, SE = 0.05). A similar pattern was observed for well‐being, where the participants scored a mean standard score of 1.66 ± 0.39 (95% CI: 1.59, 1.72). As for the professional self‐concept of participating nurses, they recorded an average mean standard score of 5.86 ± 1.64 (95% CI: 5.58, 6.13), and they scored an above average mean standard score of 3.18 ± 0.65 (95% CI: 3.07, 3.28) on the level of the empathy scale (Table 2). The relatively narrow confidence intervals indicate precise estimation of the population means.

**TABLE 2:** Total scores of study variables.

	Min	Max	Mean	SD	95% CI	SE
Spiritual intelligence	0.18	3.79	2.40	0.63	2.29, 2.50	0.05
Wellbeing	0.00	2.94	1.66	0.39	1.59, 1.72	0.03
Professional self‐concept	1.00	8.00	5.86	1.64	5.58, 6.13	0.14
Empathy	1.00	5.00	3.18	0.65	3.07, 3.28	0.05

### 5.3 Inferential Statistics

#### 5.3.1 Differences in Main Variables Based on Participants Demographics

An independent *t*‐test was performed to examine differences in the main study variables based on gender, with Levene's test confirming equal variances for all variables. The results revealed a significant gender difference in professional self‐concept (*p* < 0.001), where female nurses (*M* = 85.05, SD = 21.44) scored higher than males (*M* = 72.86, SD = 25.22). ANOVA tests were conducted to assess associations between age, religion, educational level, work unit and years of experience with the study variables. While no significant differences were found across age and religion groups, educational level showed a significant effect on spiritual intelligence (*p* = 0.01), with nurses holding master's degrees scoring higher than those with bachelor's or BT degrees. Work unit was significantly associated with professional self‐concept (*p* = 0.04), with oncology nurses scoring higher than those in medical and surgical floors. Since homogeneity of variance was violated for years of experience, a Welch *F*‐test was used, revealing a significant difference in professional self‐concept (*p* = 0.01), with more experienced nurses scoring higher. Games‐Howell post hoc tests identified the specific group differences for educational level, work unit and years of experience, Table 3.

**TABLE 3:** Demographic associations with study variables.

Demographic (test)	Main variable^a^	Statistic	*p*
Gender (*t*‐test)	Professional self‐concept	*t* = −2.80	< 0.001
	Spiritual intelligence	*t* = −0.66	0.51
	Well‐being	*t* = −0.58	0.56
	Empathy	*t* = −0.61	0.47
Age (ANOVA)	All	*F* = 0.20–2.27	0.07–0.94
Religion (ANOVA)	All	*F* = 0.06–1.20	0.32–0.99
Education (ANOVA)	Spiritual intelligence	*F* = 4.12	0.01
	Others	*F* = 0.45–2.12	0.10–0.72
Work unit (ANOVA)	Professional self‐concept	*F* = 2.18	0.04
	Others	*F* = 0.58–1.07	0.38–0.77
Experience (Welch F)	Professional self‐concept	*F* = 9.44	0.01
	Others	*F* = 0.06–2.74	0.17–0.98

^a^ Main variables: professional self‐concept, spiritual intelligence, well‐being and empathy.

Pearson correlations showed moderate positive associations between spiritual intelligence and well‐being (*r* = 0.53, *p* < 0.01), professional self‐concept (*r* = 0.54, *p* < 0.01) and empathy (*r* = 0.51, *p* < 0.01) (Table 4). In addition, well‐being showed a weak positive correlation with professional self‐concept and a moderate positive correlation with empathy (both *p* < 0.01), while empathy showed a weak positive correlation with professional self‐concept (*p* < 0.01) (Table 4).

**TABLE 4:** Correlations between study variables.

		Spiritual intelligence	Wellbeing	Professional self‐concept	Empathy
Spiritual intelligence	Pearson correlation	1.00	0.53	0.54	0.51
	Sig. (2‐tailed)		< 0.01	< 0.01	< 0.01
	*N*	143.00	143.00	143.00	143.00
Wellbeing	Pearson correlation	0.53	1.00	0.42	0.52
	Sig. (2‐tailed)	< 0.01		< 0.01	< 0.01
	*N*	143.00	143.00	143.00	143.00
Professional self‐concept	Pearson correlation	0.54	0.42	1.00	0.43
	Sig. (2‐tailed)	< 0.01	< 0.01		< 0.01
	*N*	143.00	143.00	143.00	143.00
Empathy	Pearson correlation	0.51	0.52	0.43	1.00
	Sig. (2‐tailed)	< 0.01	< 0.01	< 0.01	
	*N*	143.00	143.00	143.00	143.00

Multiple structural equation models were conducted based on previous results to examine the relationships among the study variables, revealing significant pathways between all variables. Table 5 presents unstandardized path coefficients (*B*, SE, 95% CI, p) from the structural model.

**TABLE 5:** Path coefficients from the structural model (unstandardized B).

Path	B	SE	95% CI	*p*
Professional self‐concept → empathy	0.20	0.05	0.10–0.30	< 0.001
Professional self‐concept → wellbeing	0.14	0.05	0.04–0.24	0.006
Wellbeing → professional self‐concept	0.60	0.15	0.30–0.90	< 0.001
Wellbeing → empathy	0.50	0.10	0.30–0.70	< 0.001
Spiritual intelligence → wellbeing	0.40	0.06	0.28–0.52	< 0.001
Spiritual intelligence → professional self‐concept	0.55	0.08	0.39–0.71	< 0.001
Spiritual intelligence → empathy	0.35	0.07	0.21–0.49	< 0.001

Table 6 presents the standardized direct, indirect (mediated via spiritual intelligence) and total effects for key paths. First, professional self‐concept did not demonstrate statistically significant direct effects on either empathy (*β* = 0.08, *p* = 0.12) or well‐being (*β* = 0.05, *p* = 0.18). However, it exhibited significant indirect effects on both empathy (*β* = 0.42, *p* < 0.001) and well‐being (*β* = 0.10, *p* = 0.002), resulting in significant total effects (*β* = 0.50, *p* < 0.001; *β* = 0.15, *p* = 0.004, respectively). This pattern suggests that the influence of professional self‐concept on these outcomes operates primarily through mediated pathways, rather than direct associations.

**TABLE 6:** Direct, indirect and total effects (standardized β).

Path	Direct *β* (*p*)	Indirect *β* (*p*)	Total *β* (*p*)
Professional self‐concept → empathy	0.08 (0.12)	0.42 (< 0.001)	0.50 (< 0.001)
Professional self‐concept → wellbeing	0.05 (0.18)	0.10 (0.002)	0.15 (0.004)
Wellbeing → professional self‐concept	0.10 (0.15)	0.30 (< 0.001)	0.40 (< 0.001)
Wellbeing → empathy	0.45 (< 0.001)	0.20 (< 0.001)	0.65 (< 0.001)
Spiritual intelligence → wellbeing	0.20 (< 0.001)	0.30 (< 0.001)	0.50 (< 0.001)
Spiritual intelligence → professional self‐concept	0.50 (< 0.001)	0.20 (< 0.001)	0.70 (< 0.001)
Spiritual intelligence → empathy	0.25 (< 0.001)	0.25 (< 0.001)	0.50 (< 0.001)

Similarly, well‐being did not have a significant direct effect on professional self‐concept (*β* = 0.10, *p* = 0.15), but showed a significant indirect effect (*β* = 0.30, *p* < 0.001), yielding a significant total effect (*β* = 0.40, *p* < 0.001). This further supports the presence of mediated relationships within the model.

In contrast, well‐being had a strong and significant direct effect on empathy (*β* = 0.45, *p* < 0.001), in addition to a significant indirect effect (*β* = 0.20, *p* < 0.001), resulting in a substantial total effect (*β* = 0.65, *p* < 0.001). This indicates both direct and mediated pathways linking well‐being to empathy.

Furthermore, spiritual intelligence demonstrated significant direct, indirect and total effects on all three outcome variables. It exerted a moderate direct influence on well‐being (*β* = 0.20, *p* < 0.001), professional self‐concept (*β* = 0.50, *p* < 0.001) and empathy (*β* = 0.25, *p* < 0.001), alongside additional significant indirect effects, leading to strong total effects (*β* ranging from 0.50 to 0.70, all *p* < 0.001). These findings suggest that spiritual intelligence plays a central and pervasive role within the model, influencing both intrapersonal and interpersonal outcomes through multiple pathways.

**FIGURE 1:**
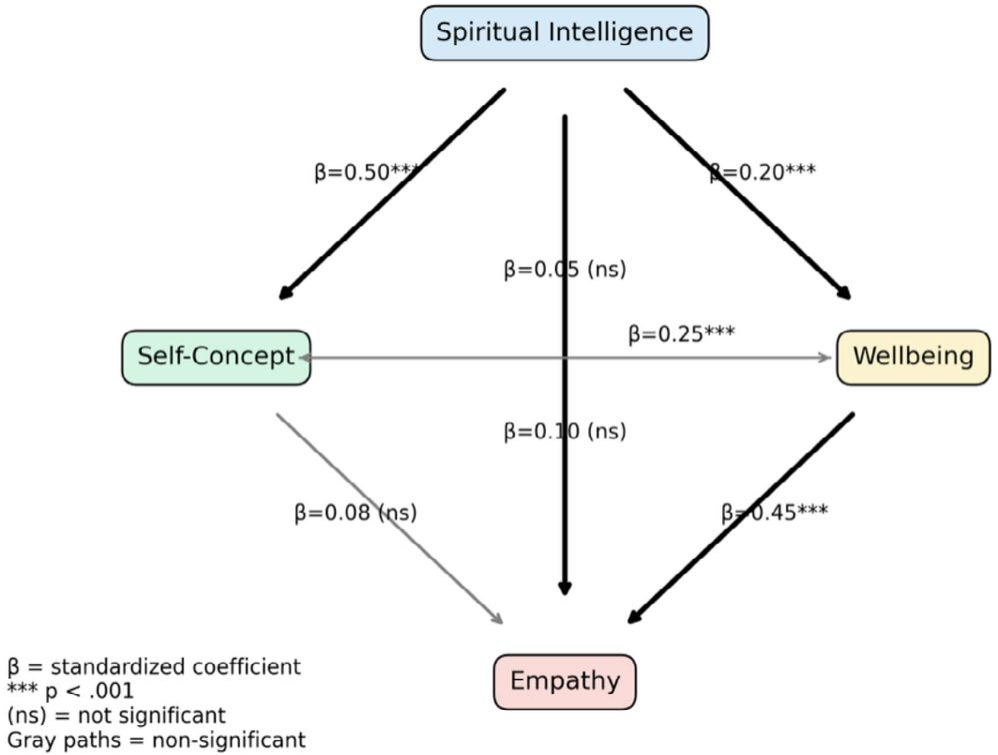
Standardized SEM path model for spiritual intelligence mediation.

Figure 1 illustrates the final SEM path model, showing standardized coefficients (*β*) for direct paths, indirect effects via spiritual intelligence and significance.

## 6 Discussion

This study examined the associations of spiritual intelligence with professional self‐concept, empathy and sense of well‐being in nurses. In recent years, growing interest has emerged in examining spirituality in healthcare and its potential influence on the nursing profession. Our study findings demonstrated that spiritual intelligence was significantly associated with nurses' well‐being scores. Well‐being is a multidimensional construct that entails factors that touch on the physical, emotional and spiritual well‐being. A connection between spiritual intelligence and the well‐being of nurses has become the focus of a few recent studies. In line with our results, researchers have found a meaningful positive correlation between the scores of spiritual intelligence and well‐being indicators of nurses (Alrashidi et al. 2022). Nurses with higher spiritual intelligence reported lower stress, greater job satisfaction, emotional stability and a stronger sense of meaning at work, positively influencing psychological outcomes. A study reported that nurses who followed spiritual care processes developed personal growth and inner peace, which made them feel better (Sharifnia et al. 2022a). These results indicate the importance of considering spiritual care as part of nursing practice to enhance the well‐being of nurses.

Nursing professionals' perceptions of themselves as capable and empathetic carers are intimately related to their professional self‐concept. According to the results of our study, spiritual intelligence showed significant associations with professional self‐concept. Pinto and Pinto (2020) conducted a study to establish how spiritual intelligence training impacts the perception of nurses towards their professional identities. The results indicated that the group of nurses that was instructed in spiritual intelligence had a stronger belief in their ability to provide caring and patient‐centred care. This enhanced self‐perception was associated with improved performance at the workplace and better relationships with patients and colleagues. Ahmadi et al. (2021) also investigated the connection between spiritual intelligence and the level of consciousness and understanding of the role of a nurse in the professional environment. According to these findings, nurses who had higher scores on the spiritual intelligence scale ranked higher on self‐awareness and better insight into their role as caretakers. This increased self‐awareness enabled nurses to practise more authentically in their work and this made them more committed to the job and more satisfied with their work.

Moreover, nurses require empathy to connect with patients personally, offering reassurance and support as needed. Recent literature has consistently shown that there is a positive relationship between spiritual intelligence and empathy in the nursing practice. The correlation between spiritual intelligence and empathy among nurses supported our results as Aliabadi et al. (2021) conducted cross‐sectional research to investigate this association. The findings revealed that nurses who reported higher spiritual intelligence scores had a greater understanding of the needs and emotions of the patients under their care. In this work, it is proposed that spiritual intelligence associates with caring patient–nurse relationships. Extending these findings, Sharifnia et al. (2022b) demonstrated that spiritual intelligence training was associated with higher empathy scores among nurses. Their findings suggested that nurses who completed this training exhibited greater emotional awareness and sensitivity, thereby enhancing their ability to understand and empathize with patients. These findings highlight the value of incorporating spiritual intelligence into nursing curricula and training programmes to strengthen nurses' empathy.

Moreover, path analysis indicated that spiritual intelligence may underlie the relationships among well‐being, professional self‐concept and empathy. This potential mediating role is consistent with prior research highlighting the role of spiritual intelligence in healthcare settings. For instance, Boudlaie et al. (2022) found that spiritual intelligence significantly predicts self‐esteem among healthcare workers and Muslim populations, supporting SI's capacity to enhance key psychological outcomes observed in our nurse sample. Furthermore, a research project by Alrashidi et al. (2022) on geriatrics discovered that spiritual intelligence accounted for associations between religion and empathy. Greater compassionate cognition was seen in older individuals who scored higher on religiousness scales, which has been partly linked to the growth of spiritual intelligence. Yet, in our study, religion played no role in regulating the examined associations. Sunaryo et al. (2017) reported that spiritual intelligence links social support and professional self‐concept among nurses. Nurses who felt greater social support indicated having an improved professional self‐concept, which was partly related to the growth of their spiritual intelligence.

The growing body of evidence demonstrating the positive influence of spiritual intelligence on nurses' well‐being, professional self‐concept and empathy further supports the findings of the present study. For example, Kaur et al. (2013) noted that spiritual intelligence could reduce nurse burnout and improve caring behaviour of nurses. Additionally, a study addressing the association between nurses' spiritual intelligence and compassion fatigue was done by Snelgar et al. (2017). The findings showed a protecting impact of spiritual intelligence against psychological exhaustion in the nursing line of work, as nurses with greater spiritual intelligence scores experienced lower levels of compassion fatigue. In another study, Kaur et al. (2015) examined the effect of spiritual intelligence on nurse–patient interactions and patient experiences. Their findings showed that nursing professionals with higher spiritual intelligence scores communicated more effectively with patients, which was associated with higher levels of patient satisfaction.

## 7 Limitations

The cross‐sectional design of this study precludes causal inferences regarding the relationships among the study variables. In addition, the use of self‐report measures may also be susceptible to social desirability bias, in which participants favour socially desirable over accurate responses. While SEM mitigated some method effects in this study, future research using objective performance measures could further minimize subjectivity and clarify variable relationships. Lastly, the limited number of previous studies examining these variables makes it harder to compare our results to what exists in the body of literature.

## 8 Conclusion and Recommendations

This study indicates spiritual intelligence associates with pathways between nurses' well‐being, professional self‐concept and empathy. Improved well‐being, a good professional self‐concept and more patient empathy were all associated with higher spiritual intelligence levels. Evidence from recent studies further supports the potential mediating role of spiritual intelligence across diverse populations, reinforcing the findings of the present study. Therefore, nursing students need to be exposed to courses or seminars that are focused on strengthening spiritual intelligence and its associations with these outcomes. Self‐awareness activities, reflective practice and discussions about the role of spirituality in patient care may all be part of workshops. Nursing academies should integrate spiritual intelligence training into curricula, equipping students with skills to deliver holistic patient care while cultivating their own spiritual intelligence competencies. Moreover, healthcare organizations should prioritize the integration of spiritual care into patient care processes. By providing patients with an opportunity to discuss their spiritual attitudes and issues with medical practitioners, this may contribute to a more patient‐centred approach to care. Nursing staff members need training on how to professionally and humanly respond to the spiritual needs of patients in order to establish a therapeutic relationship. We recommend that healthcare leaders consider evidence‐based interventions to promote spiritual intelligence development among nurses, informed by these findings. These interventions may focus on spirituality and its relevance to nursing practice through guided reflection, group discussions and peer‐support groups. Lastly, more research needs to be done regarding the impact of spiritual intelligence on nursing outcomes. Subsequent longitudinal studies should examine whether these cross‐sectional associations persist over time, particularly the relationships between spiritual intelligence training and nurses' professional self‐concept, empathy and overall well‐being. Further research examining the relationships among spiritual intelligence, cultural diversity and patient outcomes may provide valuable insights into how care can be better tailored to meet the diverse needs of patients.

## Author Contributions

All authors contributed to the study conception and design. Material preparation, data collection and analysis were primarily performed by Mirna Fawaz and Mohamad El Masri. The first draft of the manuscript was written by Ahmad Rayan. All authors reviewed and commented on previous versions of the manuscript. All authors read and approved the final manuscript.

## Funding

This research was funded by the Deanship of Scientific Research at Zarqa University, Jordan. The authors extend their appreciation to the Deanship of Scientific Research at Northern Border University, Arar, KSA, for funding this research work ‘through the project number “NBU‐FFR‐2026‐112‐02”’.

## Ethics Statement

Informed consent was obtained from all subjects and all study protocols were approved by the Institutional Review Board (IRB) at Beirut Arab University and the participating university hospital. This study adhered to the principles of the Declaration of Helsinki.

## Consent

All participants provided their consent to participate in the study.

## Conflicts of Interest

The authors declare no conflicts of interest.

## Data Availability

The data that support the findings of this study are available from the corresponding author upon reasonable request.
